# Supplementary material for: Perceived family support status and associated factors among people with hypertension in Nekemte City public hospitals, Western Ethiopia
Source: PLoS One. 2025 May 14;20(5):e0321156. doi: 10.1371/journal.pone.0321156 (PMC12077712; doi:10.1371/journal.pone.0321156)
Supplement: S1 File — (DOCX) [file pone.0321156.s001.docx]

# ENGLISH VERSION QUESTIONNAIRE

**Instructions for part I and II:** Please encircle the response of the participant among the given alternatives and for those blank spaces fill it.

P**ART I. Socio-demographic characteristics of the respondents.**

| S.No | Variables | Category |
| --- | --- | --- |
| 1 | Age | ____________years |
| 2 | Gender | 1. Male  2. Female |
| 3 | Marital status | 1. Married  2. Single  3. Divorced/separated  4. Widowed |
| 4 | Education status | 1. No formal education  2. Primary ( ≤ 8 grade )  3. Secondary (grade 9-12)  4. Higher education (TVET level and above) |
| 5 | Occupation status | 1. Employed  2 .Laborer  3. Merchants  4. Farmer  5. other (specify----------------------------) |
| 6 | Place of residence | 1.Urban  2.Rural |
| 7 | Monthly income | _______________ ETB (**Estimated**)  ­­ |
| 8 | Family sizes | 1. 2  2. 3  3. 4  4. 5 and above |

**Part II: Family-related characteristics**

| S.No | Variables | Category |
| --- | --- | --- |
| 9 | Did you have family history of hypertension? | 1.Yes  2. No |
| 10 | What is income of your family primary caregiver ? | ____________ ETB (**Estimated)** |
| 11 | What is education status of your family primary caregiver? | 1. No formal education  2. Primary( ≤ 8 grade)  3. Secondary (grade 9-12)  4. Higher education (TVET level and above). |
| 12 | Occupation of your family primary caregiver? | 1. Employed  2 .Laborer  3. Merchants  4. Farmer  5. Others ( specify________________________) |
| 13 | Gender of your family primary caregiver? | 1.Male  2.Female |
| 14 | Marital status of your family primary caregiver? | 1. Married  2. Single  3 Divorced/separated  4. Widowed |
| 15 | Did your families involved during patient education on HTN at hospital? | 1.Yes  2. No |

**Instructions for part III and IV.**

Please encircle the response of the participant among the given alternatives, for those blank spaces fill it, and make mark symbol (✓) for Likert scale .

**Part III: Patient-related characteristics**

| S.No | Variables | Category |
| --- | --- | --- |
| 16 | Did you disclose your HTN to your family? | 1.Yes  2.No |
| 17 | How many months or years since you diagnosed for HTN? | ______months or _______years |
| 18 | Did you have chronic comorbidity other than HTN? | 1.Yes  2. No |
| 19 | If response for question number 18 is yes, what are/is those/this comorbidity? | A. Diabetic mellitus  B. Heart failure  C. Renal failure  D. Other, specify_________ |
|  | Family interaction questions by using  1.strongly disagree 2.disagree 3.neutral 4.agree 5.strongly agree   \| S.No \| Variables \| category \|  \|  \|  \|  \|  \| \| --- \| --- \| --- \| --- \| --- \| --- \| --- \| --- \| \| 5 \| 4 \| 3 \| 2 \| 1 \|  \| \| 20 \| Be satisfied with communication \|  \|  \|  \|  \|  \|  \| \| 21 \| Be good listener \|  \|  \|  \|  \|  \|  \| \| 22 \| Express affection \|  \|  \|  \|  \|  \|  \| \| 23 \| Ask each other for what you want \|  \|  \|  \|  \|  \|  \| \| 24 \| Calmly discuss problems \|  \|  \|  \|  \|  \|  \| \| 25 \| Discuss ideas and beliefs \|  \|  \|  \|  \|  \|  \| \| 26 \| Ask questions and get honest answers \|  \|  \|  \|  \|  \|  \| \| 27 \| Understand feelings \|  \|  \|  \|  \|  \|  \| \| 28 \| Seldom say negative things \|  \|  \|  \|  \|  \|  \| \| 29 \| Express true feelings \|  \|  \|  \|  \|  \|  \| |  |
|  | Patients knowledge of hypertension, Assessed by using hypertension knowledge scale   \| S.No \| Item \| Yes \| No \| I don’t know \| I don’t know \| \| --- \| --- \| --- \| --- \| --- \| --- \| \| 30 \| Drugs for increased blood pressure must be taken every day. \|  \|  \|  \|  \| \| 31 \| Individuals with increased blood pressure must take their medication only when they feel ill \|  \|  \|  \|  \| \| 32 \| Individuals with increased blood pressure must take their medication throughout their life \|  \|  \|  \|  \| \| 33 \| Individuals with increased blood pressure must take their medication in a manner that makes them feel good \|  \|  \|  \|  \| \| 34 \| If the medication for increased blood pressure can control blood pressure, there is no need to change lifestyles. \|  \|  \|  \|  \| \| 35 \| Increased blood pressure is the result of aging, so treatment is unnecessary. \|  \|  \|  \|  \| \| 36 \| If individuals with increased blood pressure change their lifestyles, there is no need for treatment \|  \|  \|  \|  \| \| 37 \| Individuals with increased blood pressure can eat salty foods as long as they take their drugs regularly. \|  \|  \|  \|  \| \| 38 \| Individuals with increased blood pressure can drink alcoholic beverages \|  \|  \|  \|  \| \| 39 \| Individuals with increased blood pressure must not smoke \|  \|  \|  \|  \| \| 40 \| Individuals with increased blood pressure must eat fruits and vegetables frequently \|  \|  \|  \|  \| \| 41 \| The best type of meat for individuals with increased blood pressure is white meat. \|  \|  \|  \|  \| \| 42 \| The best type of meat for individuals with increased blood pressure is red meat. \|  \|  \|  \|  \| \| 43 \| Increased blood pressure can cause premature death if left untreated \|  \|  \|  \|  \| \| 44 \| Increased blood pressure can cause heart diseases, such as heart attack, if left untreated. \|  \|  \|  \|  \| \| 45 \| Increased blood pressure can cause strokes, if left untreated. \|  \|  \|  \|  \| \| 46 \| Increased blood pressure can cause kidney failure, if left untreated \|  \|  \|  \|  \| \| 47 \| Increased blood pressure can cause visual disturbances, if left untreated. \|  \|  \|  \|  \| |  |

**Part IV .The Perceived Social Support Family-Scale (PSS-Fa)**

| S.No | Item | Yes | No | I don’t know |
| --- | --- | --- | --- | --- |
| 48 | My families give me the moral support I need |  |  |  |
| 49 | Most other people are closer to their families than I am |  |  |  |
| 50 | My families enjoy hearing about what I think |  |  |  |
| 51 | Certain families come to me when they have problems or need advice |  |  |  |
| 52 | I rely on my families for emotional support |  |  |  |
| 53 | If I felt that one or more of my families were upset with me , I would just keep it to my self |  |  |  |
| 54 | I feel that I am on the fringe in my circle of families |  |  |  |
| 55 | There is a families I could go to if I were just feeling down, without feeling funny about it latter |  |  |  |
| 56 | My families and I are very open about what we think about things |  |  |  |
| 57 | My families are sensitive to my personal needs |  |  |  |
| 58 | My families come to me for emotional support |  |  |  |
| 59 | My families are good at helping me solve problems |  |  |  |
| 60 | I have a deep sharing relationship with a number of families |  |  |  |
| 61 | My families gets good ideas about how to do things or make things from me. |  |  |  |
| 62 | When I confide in families it makes me feel uncomfortable |  |  |  |
| 63 | My families sick me out for companionship |  |  |  |
| 64 | I think that my families feel that I am good at helping them solve problems |  |  |  |
| 65 | I don’t have a relationship with a families that is as intimate as other people s relationship with families |  |  |  |
| 66 | I have recently gotten a good idea about how to do something from a families |  |  |  |
| 67 | I wish my families were much different |  |  |  |
