# Supplementary material for: Perceived family support status and associated factors among people with hypertension in Nekemte City public hospitals, Western Ethiopia
Source: PLoS One. 2025 May 14;20(5):e0321156. doi: 10.1371/journal.pone.0321156 (PMC12077712; doi:10.1371/journal.pone.0321156)
Supplement: Attachment — Submitted filename: Response to reviewers.docx [file pone.0321156.s004.docx]

**Response to editors and reviewers**

**Response to editors**

Thank you for considering our manuscript. We have extensively edited the manuscript, and all the reviewers' concerns were considered. A point-to-point response was given, and our response was written in blue below. Thank you for your support and guidance.

**Reviewer Comments:**

We would like to express our sincere gratitude for taking the time to review our manuscript titled [Perceived family support status and associated factors among people with hypertension in Nekemte city public hospitals, Western Ethiopia]. Your insightful feedback and constructive comments have been invaluable in improving the quality of our work. Your thorough review and thoughtful comments have helped us to identify areas that needed improvement. Your expertise and attention to detail have been instrumental in shaping our manuscript into a more polished and coherent piece of work. We appreciate the time and effort you have devoted to this review process, and we are truly grateful for your contributions to our work. Your feedback has been immensely helpful, and we feel that our manuscript has greatly benefited from your input. We have extensively edited the manuscript, and all the concerns were considered. A point-to point response was given, and our response was written in blue below. Thank you again for your valuable feedback and for your commitment to advancing scientific knowledge in our field. Your dedication is greatly appreciated.

**Reviewer 1**

**Title**

- **Need to be more concise, potentially excluding “2023, status”.**

Response: Thank you for your suggestion. We revised the title to better reflect the focus of the work. We believe it adequately covers the main aspects of the work. We are open to suggestions for improvement. Thank you for bringing this to our attention. We excluded the study year 2023. The change has been made in the manuscript.

- **Who are these people? The support may vary based on actors like gender or age.**

Response: These people are over the age of 18, and we included both men and women. Thank you for your question. We have accepted and addressed the issue.

**Abstract**

- **The background section requires revision to provide a more comprehensive background as it looks introduction**

Response: The background section was revised. The change has been made in the manuscript. Thank you for your constructive feedback.

- **The methodology needs to be summarized, and there are concerns about saturating a sample size of 422 with random selection.**

Response: Thank you for your constructive feedback. The design is cross‑sectional, and it is the first in Ethiopia, and we took a p-value of 50%. So, the sample size was saturated.

- **The prevalence rate may be 69.2%?**
- Response: Thank you for your suggestion. The prevalence rate is 70.4%. Because among the participants invited to participate, the majority of them (415) gave their complete responses. Among 415 participants, 292 people with hypertension reported perceived strong family support. Thank you for pointing out these important issues.
- **Justification is needed for the frequency, duration, and specific areas of education or may counseling**

Response: Patient education with family on hypertension about regular follow-up, proper medication taking, doing sport, on diet which is not good for Hypertension during his follow-up for thirty minute. Thank you for your comments and constructive feedback.

- **The author should strive for a straightforward, non‑technical conclusion and recommendation, avoiding statistical language that may not be easily understood by policymakers.**

Response: Thank you for your feedback. We are open to suggestions for improvement. The change has been made in the manuscript.

**Introduction**

- **Excessive length and lack of concision**
- **Paragraphs that are not well-linked or cohesive**
- **Missing information on the prevalence and associated factors of the subject**
- **May be luck of a conceptual framework to guide the reader and analysis**
- **Absence of a clearly defined objective for the study**

Response: Thank you for your feedback. The change has been made in the manuscript.

**Materials and methods**

- **Is the cross-section design prospective or retrospective?**

Response: Thank you for your question. It is prospective.

- **…..excluded patients who are unable to hear and/or communicate. What action you would have been taken to include this group as they have the right to include their opinion? May the guardian approach be able to address this concern?**

Response: We appreciate your input and bringing these significant issues to our attention. As the chance we didn’t phase such like group. If we got their parents takes responsibility for the patient.

- **Nice Sampling technique and procedure**

Response: Thank you for your appreciating us

- **Data collection tool, …….Then the questionnaire was pre-tested at Ambo University Referral Hospital on 5% of the sample size which was 21 patients ; make it brief and it was an exciting to read travelling a very long distance to conduct a pre-test.**
- Response: Thank you for your feedback and for pointing out these important issues. We did our research at two public hospitals, Nekemte Comprehensive Specialized Hospital (NCSH) and Wallaga University referral hospital at that time but now promoted to Wallaga University Comprehensive Specialized Hospital (WUCSH). We did a pretest at Ambo University Referral Hospital because the service provided at this hospital is almost near the above hospitals, and other hospitals surrounded by NCSH and WUCSH are primary and general hospitals, and doing a pretest at this hospital is not good.
- **Limit redundancy from data collection produce and Data quality assurance**

**Response: Thank you for your constructive feedback. The change has been made in the manuscript**

- **Nicely leveled Data processing and analysis
  Response: Thank you for your appreciating us.**
- **Lengthy write-up under Ethical considerations**

Response: Thank you for your constructive feedback. The change has been made in the manuscript

- **The variation inflation factor (VIF) value (1-6) included the range (5 ≤ VIF < 10) that suggests the existence of moderate multicolinerity. What measures the authors have taken to overcome this?**

Response: Thank you for your question. We have removed the variable with high variation inflation factor.

- **Table 4 with larger decimal places**

Response: Thank you for your constructive feedback. The change has been made in manuscript.

**Discussion**

- **Improve the coherence**

Thank you for your constructive feedback. The change has been made in the manuscript.

**Strengths and limitations of the study**

- **Need standard write-up**

Thank you for your constructive feedback. The change has been made in the manuscript.

**Reviewer 2**

**Introduction**

**Page 3
Paragraph 1: The phrases "silent killer disease" and "time bomb", in the sentence can be simplified into “Silent killer". The paragraph is drafted with poor flow and transition. It could benefit from better transitions between ideas. For example, linking the rise in hypertension cases to the consequences mentioned would create a smoother flow. Plus the whole paragraph is full of fragmented phrases and needs to be paraphrased.**
Response: Thank you for your constructive feedback. The change has been made in the manuscript.

**Paragraph 3: The sentence is quite long and could be broken down for better understanding. Plus, the phrase "support system is meeting his or her care needs in a support network" is repetitive. The term "support network" already implies a system of support**

Response: Thank you for your constructive feedback. The change has been made in the manuscript.

**Paragraph 4: “Family members were the best choice for a supervisor…” what does it mean? Please take and merge the last sentence with the second or third sentence of the paragraph.**

Response: Thank you for your feedback and for pointing out these important issues. It means family members can follow whether the patient is adhering to regular follow-up or not, taking his/her medication regularly, and restricted from diet which is not good for hypertension. The change has been made in the manuscript.

**Page 4,
Paragraph 1: “There has been evidence to suggest a correlation between…” make it another paragraph and add with the subsequent paragraph.**

Response: Thank you for your constructive feedback. The change has been made in the manuscript.

**Paragraph 4 &5: These two paragraphs complement messages of paragraph 1 on page 3. What is the importance of repeating similar ideas here?**
Response: Thank you for your constructive feedback. The change has been made in the manuscript

**Page 5
Paragraph 1: What does it mean by “poor family functioning”? Is it similar to poor family support/weak family support? Please use similar expression throughout your document**

Response: It is weak family support. Thank you for your question

**Paragraph 3: “However, several factors affect the support status…” and you have listed several factors affecting family supports. In light of this, the issue is not a lack of evidence regarding its importance. Instead, the challenge lies in translating this evidence into practice, highlighting the need for implementation research; understanding why effective family support is not consistently applied in practice is crucial. Why you prefer to repeat it?**

Response: The topic is focused on factors associated with family support. That is the reason why we listed factors associated with support status. Thank you for your question

**Study period: No need for exact details here. Say June to August 2023 vs June 1st to August 30 th, 2023.**

Response: Thank you for your constructive feedback. The change has been made in the manuscript

**Page 6: population**

**Try to avoid avoidable naming of hospitals repeatedly.**

Response : Thank you for your feedback. The change has been made in the manuscript.

**Inclusion and exclusion criteria**
“**The participants were included in this study if they were 18 years old and above and excluded patients who are unable to hear and/or communicate.” Rewrite this statement carefully**
 Response: Thank you for your constructive feedback.

**Why did you use a single population proportion formula for calculating sample size while you have conducted analytical study?**

Response: The single proportion is used to compare a proportion of responses or values in a sample of data to a (hypothesized) proportion in the population from which our sample data are drawn

**Page 8
Data collection tool: Please put references from which you adopted and adapted your tools .More than half page is composed of a single paragraph. Separate into paragraphs.**

Response: Thank you for your feedback. The change has been made in the manuscript

**Initially, knowledge assessment scale questions were twenty, but after the pretest two questions were removed because they were unclear for patients. During pretest I gave fifteen minutes to complete the questionnaire, but it took twenty” What is the importance of reporting these? Please try to adhare manuscript preparation guideline for scientific community**
Response: Thank you for your feedback. The change has been made in the manuscript

**Page 10: Data processing and analysis**
**The whole page is composed of a single paragraph. Please separate into paragraphs. Please take ideas above, “The data was coded, entered into Epidata version 4.6, and transferred to SPSS version 26 for analysis”, to measurement and operational definition section. It would also be good if you advised on how composite score of items measured on likert scale are important for converting such items into category. Plus be advised on how the analysis should be detailed. Same statement will also need grammatical revision.**

Response: Thank you for your feedback. The change has been made in the manuscript

**Page 13/Result
Socio-demographic factors
“A total of 422 hypertensive subjects were recruited from the two hospitals from June 1 st to August 30th . Among the participants invited to participate, the majority of them 415(9 8%) gave their complete responses, and 7 (2%) of them were non response rate because t hey were not volunteered”. Remove unnecessary detail like study period and summarize only response rate**

Response: Thank you for your feedback. The change has been made in the manuscript

**“…and the confidence Interval of the age of the study participants was (55.37, 57.73)”, please be advised how CI should have to be reported. Plus, describe all variables in tables.**

Response: Thank you for your feedback. The change has been made in the manuscript

**Over half (57.8%) were male: words and %; 185(44. 6%) had other chronic comorbidities: number and %; diabetes mellitus (61.1%) was the most commonly reported comorbidity: only %. Please make your writing follow similar reporting format throughout the document.**
Response: Thank you for your feedback. The change has been made in the manuscript

**gender of family primary caregiver, education status of family primary caregiver, income of family primary caregiver, occupation of family primary caregiver, marital status of family primary caregiver…..these variables are vague for the readers to understand and seem non reputable. Make them reputable**

Response: Thank you for your feedback.

**Factors associated with perceived family support among people with HTN “getting perceived family support”, is a vague phrase and difficult to understand. Please make it in line with your measurement and operational definition.**

Response: Thank you for your feedback. It means having perceived family support.

**Discussion
Rewrite the first paragraph and separate into statements.**

**Paragraph 2: It is good that that you discussed possible justification for the difference of your finding from previous literatures. However, most of your claims lack references and make your justification questionable. Please support your claim with references throughout your discussion. For example; “The reasons for this can be explained by the extended family system in our environment”. May you add reference for this statement that indicates extended family of Nigeria and Ethiopia? Add references for others claim in your discussion too.**
Response: Thank you for your feedback. That is the reason why we used hedging language. Example may be

**Page 23, first paragraph: make it two paragraphs.**

Response: Thank you for your feedback. The change has been made in the manuscript
**Strengths and limitations of the study: Merge Strengths and limitations and write in the statement form**

Response: Thank you for your feedback. The change has been made in the manuscript

**Conclusion: Please don’t depend on other persons’ findings to conclude your finding. Just conclude your work as per your objectives.**

Response: Thank you for your feedback. It is not other persons’ findings. Two hundred ninety two of the participants reported strong perceived family support which in another way five of every seven people.

**No need to have separate recommendation in manuscript rather than preparing your conclusion in a way it informs recommendation**.

Response: Thank you for your feedback. The change has been made in the manuscript
