# Supplementary material for: Perceived family support status and associated factors among people with hypertension in Nekemte City public hospitals, Western Ethiopia
Source: PLoS One. 2025 May 14;20(5):e0321156. doi: 10.1371/journal.pone.0321156 (PMC12077712; doi:10.1371/journal.pone.0321156)
Supplement: Attachment — Submitted filename: RESPONSE_TO_REVIEWERS_auresp_2.docx [file pone.0321156.s005.docx]

**RESPONSE TO REVIEWERS**

Reviewer #1: Dear authors, your paper now looks fine for dissemination at global scale. Thank you for the revision, and again consider these minor point.

\# Include reference for this sentence in paragraph one of introduction “Effective management is important to reduce morbidity and mortality”.

**Response: Thank you for your suggestion. Since the study is not interventional, we removed this sentence. The change is made in the manuscript.**

#If you did not encounter individuals who could not hear during data collection, it would not be appropriate to list them as an exclusion criterion unless you had a specific rationale for excluding them that was relevant to your study design.

**Response: Thank you for your suggestion. We did not encounter participants who could not hear. Hence, we removed the criterion. The change is made in the manuscript.**

Reviewer #2: Authors partly modified the manuscript. However, the authors responses are too shallow and did not cover my previous comments. Some comments like research gaps, sample size calculation and measurement need justification in manuscript rather than trying to respond for the reviewers. The manuscript may be benefited from revision considering the previous review results

**Response: Thank you for your concern. The final required sample size was estimated using a single population proportion formula because the estimated sample sizes using variables found predictors from previous studies are less than the estimated sample size using a single population proportion. Hence, we used the largest sample size that is estimated using a single population proportion. The justification is given in the manuscript.**

**The research gap and the measurement is revised. The change is made in the manuscript.**
