# Supplementary material for: Perceived family support status and associated factors among people with hypertension in Nekemte City public hospitals, Western Ethiopia
Source: PLoS One. 2025 May 14;20(5):e0321156. doi: 10.1371/journal.pone.0321156 (PMC12077712; doi:10.1371/journal.pone.0321156)
Supplement: Attachment — Submitted filename: Reviewer comments.docx [file pone.0321156.s002.docx]

The document is full of grammatical illusions, and can lead readers to initially interpret them in a way that seems correct, only to reveal a grammatical error upon closer examination. The document may be benefited from major grammatical and punctuation revision. Authors have to be advised on manuscript preparation for publication, word selection and necessary detail needed in manuscript. The manuscript has no line numbers, making it difficult to make reference to sections of the document while reviewing. PDF version was also difficult to make line numbers from the reviewer's copy.

Introduction

Page 3

Paragraph 1: The phrases "silent killer disease" and "time bomb" , in the sentence can be simplified into “Silent killer". The paragraph is drafted with poor flow and transition. It could benefit from better transitions between ideas. For example, linking the rise in hypertension cases to the consequences mentioned would create a smoother flow. Plus the whole paragraph is full of fragmented phrases and needs to be paraphrased.

Paragraph 3: The sentence is quite long and could be broken down for better understanding. Plus, the phrase "support system is meeting his or her care needs in a support network" is repetitive. The term "support network" already implies a system of support

Paragraph 4: “Family members were the best choi ce for a supervisor…” what does it mean? Please take and merge the last sentence with the second or third sentence of the paragraph.

Page 4,

Paragraph 1: “There has been evidence to suggest a correlation between…” make it another paragraph and add with the subsequent paragraph.

Paragraph 4 &5: These two paragraphs complement messages of paragraph 1 on page 3. What is the importance of repeating similar ideas here?

Page 5

Paragraph 1: What does it mean by “poor family functioning”? Is it similar to poor family support/weak family support? Please use similar expression throughout your document

Paragraph 3: “However, several factors affect the support status…” and you have listed several factors affecting family supports. In light of this, the issue is not a lack of evidence regarding its importance. Instead, the challenge lies in translating this evidence into practice, highlighting the need for implementation research; understanding why effective family support is not consistently applied in practice is crucial. Why you prefer to repeat it?

Study period: No need for exact details here. Say June to August 2023 vs June 1st to August 30 th, 2023.

Page 6: population

Try to avoid avoidable naming of hospitals repeatedly.

Inclusion and exclusion criteria

“The participants were included in this study if they were 18 years old and above and excluded patients who are unable to hear and/or communicate.” Rewrite this statement carefully

Sample size determination
Why did you use a single population proportion formula for calculating sample size while you have conducted analytical study?

Please know how far detail sample size calculation and sampling technique should be in manuscript prepared for publication.

Page 8

Data collection tool: Please put references from which you adopted and adapted your tools

More than half page is composed of a single paragraph. Separate into paragraphs.

“Initially, knowledge assessment scale questions were t wenty, but after the pretest two questions were removed because they were unclear for pa tients. During pretest I gave fifteen minutes to complete the questionnaire, but it took twenty” What is the importance of reporting these? Please try to adhare manuscript preparation guideline for scientific community

Please merge and describe your study variables, operational definition and measurement well.

Page 10: Data processing and analysis

The whole page is composed of a single paragraph. Please separate into paragraphs. Please take ideas above, “The data was coded, entered into Epidata version 4.6, and transferred to SPSS version 26 for analysis”, to measurement and operational definition section. It would also be good if you advised on how composite score of items measured on likert scale are important for converting such items into category. Plus be advised on how the analysis should be detailed. Same statement will also need grammatical revision.

Page 13/Result

Socio-demographic factors

“A total of 422 hypertensive subjects were recruited from the two hospitals from June 1 st to August 30th . Among the participants invited to participate, the majority of them 415(9 8%) gave their complete responses, and 7 (2%) of them were non response rate because t hey were not volunteered”. Remove unnecessary detail like study period and summarize only response rate.

“…and the confidence Int erval of the age of the study participants was (55.37, 57.73)”, please be advised how CI should have to be reported. Plus, describe all variables in tables.

Over half (57.8%) were male: words and %; 185(44. 6%) had other chronic comorbidities: number and %; diabetes mellitus (61.1%) was the most co mmonly reported comorbidity: only %. Please make your writing follow similar reporting format throughout the document.

gender of family primary ca regiver, education status of family primary caregiver, income of family primary caregiver, occupation of family primary caregiver, marital status of family primary caregiver…..these variables are vague for the readers to understand and seem non reputable. Make them reputable.

Factors associated with perceived family support among people with HTN

“getting perceived family support”, is a vague phrase and difficult to understand. Please make it in line with your measurement and operational definition.

Discussion

Rewrite the first paragraph and separate into statements.

Paragraph 2: It is good that that you discussed possible justification for the difference of your finding from previous literatures. However, most of your claims lack references and make your justification questionable. Please support your claim with references throughout your discussion. For example; “The reasons for this can be explained by the extended family system in our environment”. May you add reference for this statement that indicates extended family of Nigeria and Ethiopia? Add references for others claim in your discussion too.

Page 23, first paragraph: make it two paragraphs.

Strengths and limitations of the study: Merge Strengths and limitations and write in the statement form

Conclusion: Please don’t depend on other persons’ findings to conclude your finding. Just conclude your work as per your objectives. No need to have separate recommendation in manuscript rather than preparing your conclusion in a way it informs recommendation.
